# Persistent Systemic Inflammation in Patients With Severe Burn Injury Is Accompanied by Influx of Immature Neutrophils and Shifts in T Cell Subsets and Cytokine Profiles

**DOI:** 10.3389/fimmu.2020.621222

**Published:** 2021-01-29

**Authors:** Patrick P. G. Mulder, Marcel Vlig, Bouke K. H. L. Boekema, Matthea M. Stoop, Anouk Pijpe, Paul P. M. van Zuijlen, Evelien de Jong, Bram van Cranenbroek, Irma Joosten, Hans J. P. M. Koenen, Magda M. W. Ulrich

**Affiliations:** ^1^Preclinical Research, Association of Dutch Burn Centres (ADBC), Beverwijk, Netherlands; ^2^Laboratory of Medical Immunology, Department of Laboratory Medicine, Radboud University Medical Center, Nijmegen, Netherlands; ^3^Burn Center, Red Cross Hospital, Beverwijk, Netherlands; ^4^Department of Plastic and Reconstructive Surgery, Red Cross Hospital, Beverwijk, Netherlands; ^5^Department of Plastic, Reconstructive and Hand Surgery, Amsterdam Movement Sciences Amsterdam UMC, Location VUmc, Amsterdam, Netherlands; ^6^Pediatric Surgical Centre, Emma Children’s Hospital, Amsterdam UMC, University of Amsterdam, Vrije Universiteit, Amsterdam, Netherlands; ^7^Department of Intensive Care, Red Cross Hospital, Beverwijk, Netherlands

**Keywords:** immune response, neutrophils, monocytes, lymphocytes, systemic, inflammation, flow cytometry, burn injury

## Abstract

Severe burn injury causes local and systemic immune responses that can persist up to months, and can lead to systemic inflammatory response syndrome, organ damage and long-term sequalae such as hypertrophic scarring. To prevent these pathological conditions, a better understanding of the underlying mechanisms is essential. In this longitudinal study, we analyzed the temporal peripheral blood immune profile of 20 burn wound patients admitted to the intensive care by flow cytometry and secretome profiling, and compared this to data from 20 healthy subjects. The patient cohort showed signs of systemic inflammation and persistently high levels of pro-inflammatory soluble mediators, such as IL-6, IL-8, MCP-1, MIP-1β, and MIP-3α, were measured. Using both unsupervised and supervised flow cytometry techniques, we observed a continuous release of neutrophils and monocytes into the blood for at least 39 days. Increased numbers of immature neutrophils were present in peripheral blood in the first three weeks after injury (0.1–2.8 × 10^6^/ml after burn vs. 5 × 10^3^/ml in healthy controls). Total lymphocyte numbers did not increase, but numbers of effector T cells as well as regulatory T cells were increased from the second week onward. Within the CD4^+^ T cell population, elevated numbers of CCR4^+^CCR6^-^ and CCR4^+^CCR6^+^ cells were found. Altogether, these data reveal that severe burn injury induced a persistent innate inflammatory response, including a release of immature neutrophils, and shifts in the T cell composition toward an overall more pro-inflammatory phenotype, thereby continuing systemic inflammation and increasing the risk of secondary complications.

## Introduction

Burn injury and its consequences affect patients’ overall health and quality of life because of long-term functional and cosmetic impairment (1). Severe burn trauma induces pro-inflammatory immune responses in peripheral blood and affected tissues, regardless of infection (2, 3). This immune response can persist up to months and can lead to additional health problems, including systemic inflammatory response syndrome (SIRS), hypermetabolic state and damage to surrounding tissues and even distant organs (4–7). Trauma instantly causes inflammation and produces damage associated molecular patterns (DAMPs) through necrotic and injured tissue, which stimulates the immune system to recruit acute phase immune cells (8, 9). A well-orchestrated immune response is essential for a proper healing process, as a persistent and dysregulated immune reaction can negatively affect wound closure and tissue repair. For example, an overactive immune system can cause tissue damage by proteases and oxygen radicals released by innate immune cells, and by hypercoagulation-induced ischemia (10, 11). Such collateral damage can even be linked to excessive scarring (12), which in turn cause debilitating deficiencies affecting physical, psychological and social aspects. So far, studies examining burn-induced systemic inflammation centered on data obtained from animal models (13). The human response to trauma is however quite distinct from that of animals, which is exemplified by differences in wound healing and scar formation (14). Data on the mechanisms behind the propagation and regulation of burn-induced immune response in humans is still very limited (15).

After initiation of the acute phase immune response due to burn injury, neutrophils and macrophages are the first immune cells homing to the wound area (1). Neutrophils and macrophages originate from the blood circulation and are replenished by the bone marrow. These innate immune cells remove necrotic tissue and defend the body from pathogens by phagocytosis and the release of reactive oxygen species (16). In this inflammatory phase, the innate immune cells enhance the inflammation and recruit other immune cells by secreting soluble mediators (3). In the late phase of inflammation, T cells, originating from lymphoid tissues, and anti-inflammatory macrophages resolve the inflammation to limit ancillary damage to the tissue (17). In trauma, T cell subtypes Th1 and Th17 cells play a role in the enhancement of inflammation, whereas Th2 and regulatory T cells (Tregs) are involved in its resolution (18). A balance between these subtypes is essential for a proper transition from inflammation to wound healing (19). In a normal wound healing situation, i.e. after minor injuries, neutrophils disappear from the wound area through apoptosis and macrophages differentiate from a pro-inflammatory state to a tissue remodeling state to re-establish homeostasis and initiate the proliferation phase wherein restoration of the skin can take place (20).

In burn trauma, the coordinated immune response is distorted and extended. A burn-induced hyperinflammatory state is accompanied by significant elevation of immune cells, cytokines, and acute phase proteins (9). Particularly serum interleukin (IL)-6, IL-8, granulocyte colony-stimulating factor and monocyte chemoattractant protein (MCP)-1 revealed dramatic increases in a large set of severely burned (pediatric) patients (5, 9). These increases in cytokine levels were dependent on the size of the injury at 24–48 h after trauma (5). In response to thermal injury, there is a rapid increase in bone marrow-derived endothelial progenitor cells in peripheral blood, which correlates with the extent of injury (21).

In order to improve wound healing and limit the formation of hypertrophic scars, an improved understanding of the immune response induced by severe trauma is needed. This knowledge, together with clinical perspectives, could be used to resolve an excessive immune response by therapy to restore the immune balance and optimize wound healing. Although the time-course of cytokines due to burns has been reported (5, 9, 22), data on immune cells were not included. Our aim was to characterize the inflammatory response by investigating peripheral blood changes in subsets of innate and adaptive immune cells in time [post burn day (PBD) 0–39] and 33 inflammatory mediators (PBD 0–48) in adult patients with severe burn injury.

## Materials and Methods

### Flow Cytometry

Plasma was separated from blood cells by centrifugation for 10 min at 450 × *g* and stored at -80 °C. Erythrocyte lysis buffer (1.5 mM NH_4_Cl, 0.1 mM NaHCO_3_ and 0.01 mM EDTA (Life Technologies, Paisley, UK) in demineralized water) was used to remove erythrocytes from the blood cells. Blood immune cells were resuspended in Dulbecco’s phosphate buffered saline (Gibco, ThermoFisher, Paisley, UK) containing 0.2 mM bovine serum albumin (Fisher Scientific, Pittsburgh, PA) and 0.01 mM EDTA. Cell concentrations were determined by a flow cytometer (MACS Quant Analyzer 10, Miltenyi Biotec GmbH, Bergisch Gladbach, Germany). Cell suspensions of 2.5 × 10^5^ cells each were stained with different antibody combinations (see panels in **Supplementary Table 2**) and were analyzed by flow cytometry (MACS Quant Analyzer 10). Samples with more than 40% dead cells [based on 7-AAD staining (Miltenyi)] were excluded from the analysis. Singlet events were gated based on FSC. Viable CD45^+^ cells were gated and subtyped based on expression of the markers in the 3 staining panels: innate panel (CD10, CD11b, CD14, CD15, and CD16), eosinophil panel (CD9, CD15, and CD16), and lymphocyte panel (CD3, CD4, CD25, CD127, CCR4/CD194, and CCR6/CD196). Manual data analysis was performed using the FlowLogic software (Inivai Technologies, Victoria, Australia).

### Gating Strategy for Supervised Flow Cytometry

The gating strategy is shown in **Supplementary Figure 1**. Viable CD45^+^ cells were gated on FSC and SSC to characterize granulocytes, monocytes and lymphocytes. Subsequently, cells were determined as follows: immature neutrophils (CD10^dim^CD15^+^CD16^+^ granulocytes), mature neutrophils (CD10^bright^CD15^+^CD16^+^ granulocytes), eosinophils (CD9^+^CD15^+^CD16^-^ granulocytes), classical monocytes (CD14^bright^CD16^-^ monocytes), intermediate monocytes (CD14^bright^CD16^+^ monocytes), non-classical monocytes (CD14^dim^CD16^+^ monocytes), T cells (CD3^+^ lymphocytes), and Tregs (CD3^+^CD25^+^CD127^-^).

### Unsupervised Analysis of Flow Cytometry Data

The innate and lymphocyte panel were used for unsupervised analysis in Cytobank (23). Viable monocytes, granulocytes or lymphocytes were gated using 7-AAD and CD45 staining and FSC/SSC in MACSQuantify 2.13 software (Miltenyi). The data was uploaded to Cytobank to create Flow Self-Organizing Map (FlowSOM) cluster plots.

### Plasma Cytokine Analysis

Plasma samples were thawed, and debris was removed using a filter plate (Multiscreen, Merck KGaA, Darmstadt, Germany). Luminex assay was performed according to the manufacturer’s instructions (Merck KGaA). The following assay kits were used: HCYTA-60K, TGFBMAG-64K, HCYTA-60K, HCYP2MAG-62K and HTH17MAG-14K. In short, 25 µL of plasma was used to determine the concentrations of 33 cytokines and chemokines, namely MCP-1 (CCL2), MIP-1α (CCL3), MIP-1β (CCL4), MIP-3α (CCL20), GRO-α (CXCL1), IP-10 (CXCL10), IFN-α2, IFN-γ, TNF-α, TGF-β1, TGF-β2, TGF-β3, CTACK (CCL27), RANTES (CCL5; in a 1:100 dilution), IL-1α, IL-1β, IL-2, IL-4, IL-5, IL-6, IL-8 (CXCL8), IL-9, IL-10, IL-12p40, IL-12p70, IL-13, IL-17A (CTLA-8), IL-17F, IL-18, IL-21, IL-22, IL-23, and IL-33 (NF-HEV). Mean fluorescence intensity of samples was measured with a Flexmap 3D System (Luminex Corp, Austin, USA) and concentrations were calculated using Bio-Plex Manager Software (Bio-Rad Laboratories, Veenendaal, The Netherlands). When cytokine levels were out of range of the standard, either the lowest level of quantification or the highest level of quantification was used. To combine results of multiple assays, we transformed the data to fold changes of healthy controls.

### Statistical Analyses

Distribution of the data was checked for normality. For the flow cytometry data, differences between the levels of outcomes of patients on PBD 0–3 and healthy controls were explored using the Mann Whitney U test. Results per time interval (e.g., PBD 0–3) were averaged per patient. Differences in outcomes within patients between time intervals PBD 4–6 through PBD 37–39 vs. PBD 0–3 were analyzed in SPSS version 25 (IBM, Armonk, USA) using linear mixed model to correct for the dependent data structure. The outcome measurements were used as the dependent variable in the models. Time was entered as a categorical variable in the model as a fixed effect. Level of statistical significance was set at p < 0.05. The data was visualized using Graphpad version 5.01 (PRISM, La Jolla, USA).

Data of the soluble immune factors was transformed to fold changes of healthy controls. P values between time intervals and healthy controls were determined using Mann Whitney U tests. Because of multiple testing, we considered a p value of <0.01 to be significant. Volcano plots were created using “EnhancedVolcano” version 1.6.0 package in R version 3.6.2.

### Subject Recruitment and Sample Collection

Twenty burn wound patients admitted to the intensive care unit (ICU) of the Burn Center of the Red Cross Hospital in Beverwijk, the Netherlands were included in this study after written consent was obtained from the patient or a legal representative (for subject details see **Supplementary Table 1A**). The study protocol with number “NL54823.094.15” was approved by the METc of the VU Medical Center (Amsterdam, the Netherlands). Patients were eligible from 18 years of age with a burned total body surface area (TBSA) of ≥ 15%. Subjects were included between April 2018 and April 2020. Venous blood samples were collected on a daily basis when present on ICU or twice per week when transferred to the infirmary. Blood samples taken from 20 healthy volunteers served as controls (METc approved under protocol number “NL54823.094.15”). Blood levels of C-reactive protein (CRP), albumin and thrombocytes were determined according to standard diagnostic laboratory procedures as part of standard burn care. Blood samples for flow cytometry were collected in ethylenediaminetetraacetic acid (EDTA) tubes and were stored at 4°C until analysis (<3 h). Only blood samples from working days were used for flow cytometric analysis. The frequency of sampling of each individual patient is presented in **Supplementary Table 1B**. The patients were treated according to standard burn care, including fluid resuscitation. All patients received analgesics (including paracetamol, NSAIDs and opiates) and antibiotics One of the included patients died two days after the trauma. Three patients contracted pneumonia, one patient had an infected hematoma and none of the patients had sepsis or full-blown infection due to their burn injuries. Colonization of burn wounds was noted, and the predominant bacterial species were *Staphylococcus aureus* (10/20), *Enterococcus cloaca* (10/20), *Pseudomonas aeruginosa* (7/20) and *Escherichia coli* (5/20).

## Results

### Systemic Inflammation After Burn Injury is Associated With Prolonged Increase of Peripheral Blood Granulocytes and Monocytes

**Figure 1:**
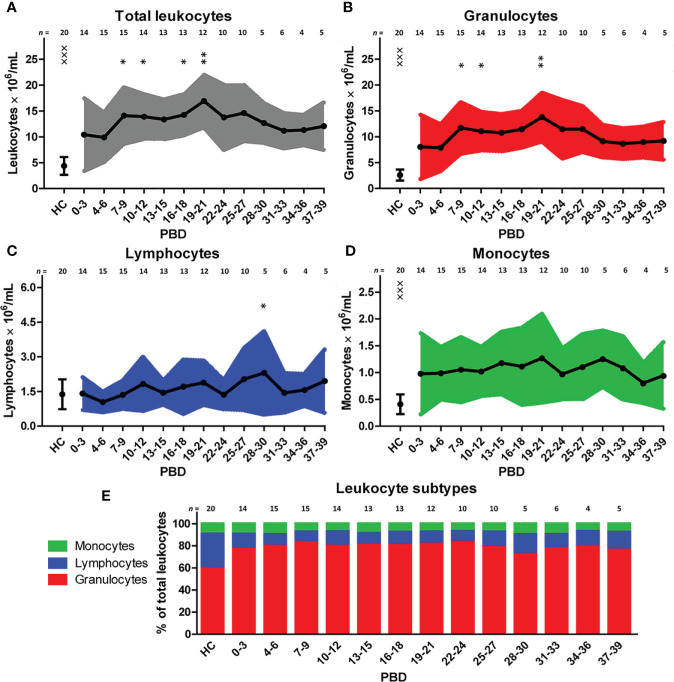
Blood immune cells after severe burn injury. Flow cytometry was used for phenotyping of leukocytes: **(A)** Total leukocyte numbers (gray). **(B)** Granulocyte numbers (red). **(C)** Lymphocyte numbers (blue). **(D)** Monocyte numbers (green). **(E)** Relative amount of leukocyte subtypes. Number of subjects per time interval is shown on top of the graphs. Values of burn wound patients and healthy controls (HC) are shown as mean (line and dots) ± standard deviation (colored band). Asterisks indicate significant differences in time within the burn patient group (linear mixed model analysis): *p < 0.05; **p < 0.01. Significant differences of outcomes in burn patients of PBD 0-3 compared to healthy controls are indicated by × (^×××^p < 0.001).

To examine the immune profile of burn wound patients in more detail, we performed multiparameter phenotyping by flow cytometry of peripheral blood of 20 burn wound patients up to 39 days after burn injury. All burn patients in the cohort showed signs of systemic inflammation (**Supplementary Figure 2**). Immediately after burn injury, total blood leukocyte counts were significantly increased compared to healthy controls. To analyze the response in time, a linear mixed model analysis was performed to determine the changes in comparison to time interval PBD 0–3. Data of PBD 0–3 was available for 15 patients. This analysis showed an additional increase in leukocyte counts until PBD 19–21 with the exception of PBD 13–15 (**Figure 1A**). Subtype analysis revealed that this increase of blood leukocytes could be ascribed to granulocyte and monocyte numbers, and not to lymphocyte counts (**Figures 1B–D**). In burn patients, granulocyte and monocyte counts rose significantly immediately after the injury compared to healthy controls but remained stable during the total time course (**Figures 1B, D**). Lymphocyte counts showed no increase compared to healthy controls. A small decrease around PBD 4–6 compared to PBD 0–3 was seen, followed by a non-significant tendency toward higher lymphocyte counts (**Figure 1C**). The relative amounts of leukocyte subtypes are summarized in **Figure 1E**. We observed no confounding effect of TBSA (> 26% vs. ≤ 26%) on the course of the inflammatory response (i.e. leukocytes, granulocytes, lymphocytes, and monocytes) in the mixed model analysis (data not shown).

### Burn Injury Is Associated With A Large, Continuous Surge of Immature Neutrophils, Classical and Non-Classical Monocytes

**Figure 2:**
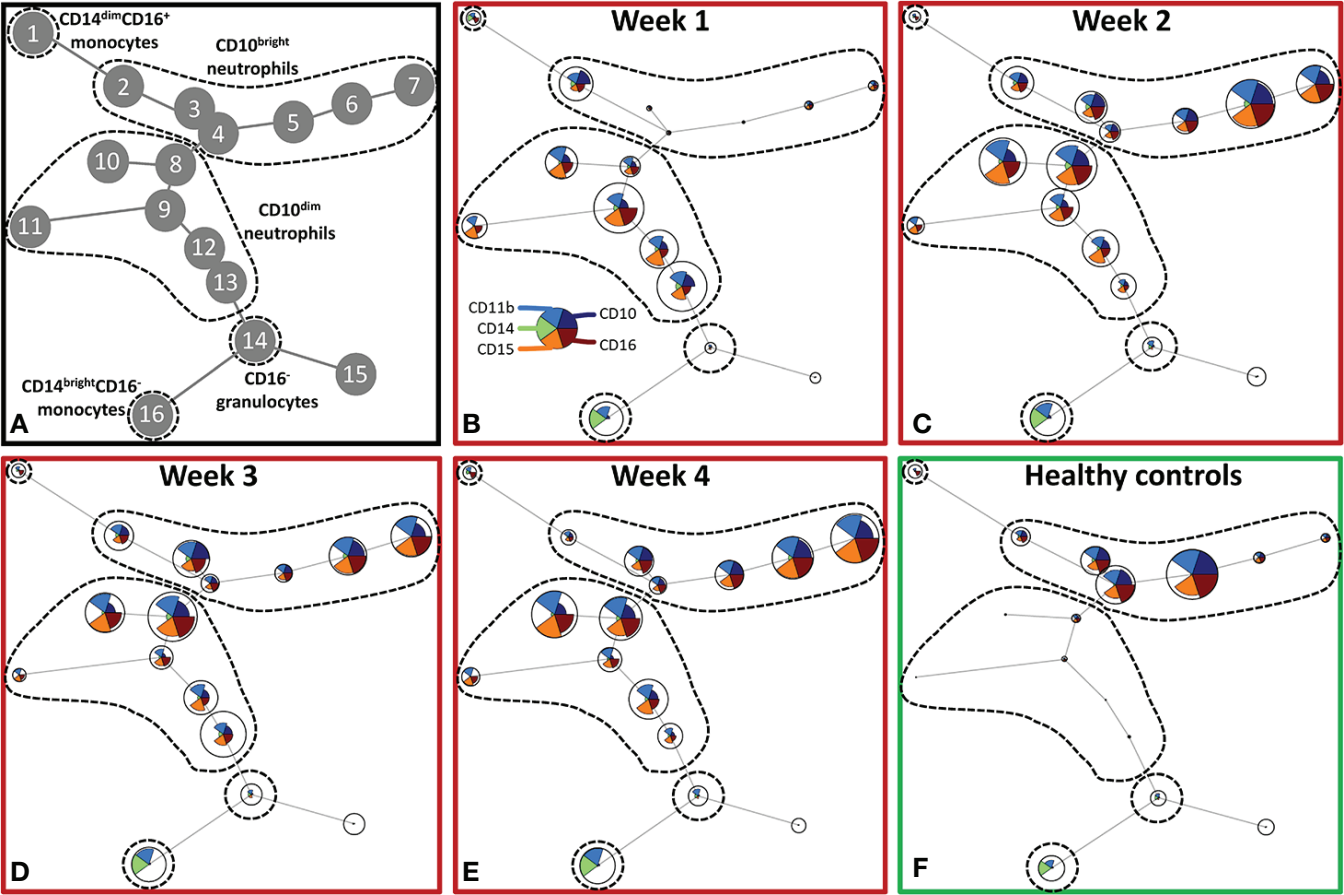
Unsupervised FlowSOM analysis of granulocyte and monocyte subtypes after severe burn injury. FlowSOM plots present proportions of populations and the expression of markers that were used in the innate flow cytometry panel (CD10, CD11b, CD14, CD15 and CD16). **(A)** Cluster structure based on flow cytometry data of 10 healthy controls and 7 burn wound patients that were observed for 4 weeks. The most pronounced subtypes are encircled by dashed lines: CD16^+^ monocytes (node 1), CD10^bright^ neutrophils (nodes 2-7), CD10^dim^ neutrophils (nodes 8-13), CD16^-^ granulocytes (node 14), CD14^dim-^ CD14^bright^CD16^-^ monocytes (node 16). FlowSOM plots of: **(B)** Week 1; **(C)** Week 2; **(D)** Week 3; **(E)** Week 4 after burn; **(F)** Healthy controls.

To further explore the effect of severe burn injury on systemic granulocyte and monocyte subsets in time, we performed an unsupervised analysis using Flow Self-Organizing Map clustering (FlowSOM) (**Figure 2**). We used data from flow cytometry stainings of 7 patients from which samples of all time points were available. The FlowSOM cluster structure was determined based on all data from these patients and 10 healthy controls. We could define 5 main cell clusters: CD10^dim^ neutrophils (nodes 8–13), CD10^bright^ neutrophils (nodes 2–7), CD16^-^ granulocytes (including eosinophils; node 14), classical CD14^bright^CD16^-^ monocytes (node 16) and non-classical CD14^dim^CD16^+^ monocytes (node 1) (**Figure 2A**). Then, we analyzed the composition of these clusters in burn patients over time. CD10 was previously associated with the maturation stages of neutrophils (24–26). In the first week post burn, the three mature (CD10^bright^) neutrophil populations (nodes 3–5) were hardly present and the majority of neutrophils was immature (CD10^dim^). From week 2 onward, mature CD10^bright^ neutrophils reappeared, while immature CD10^dim^ neutrophil numbers declined, but remained elevated for the remaining period of the study. The number of CD16^-^ granulocytes slightly decreased in week 1 and returned to the level of healthy controls in week 2. Burn injury caused a shift toward more classical CD14^bright^CD16^-^ monocytes and the elevated level of this subtype persisted for the whole study period.

**Figure 3:**
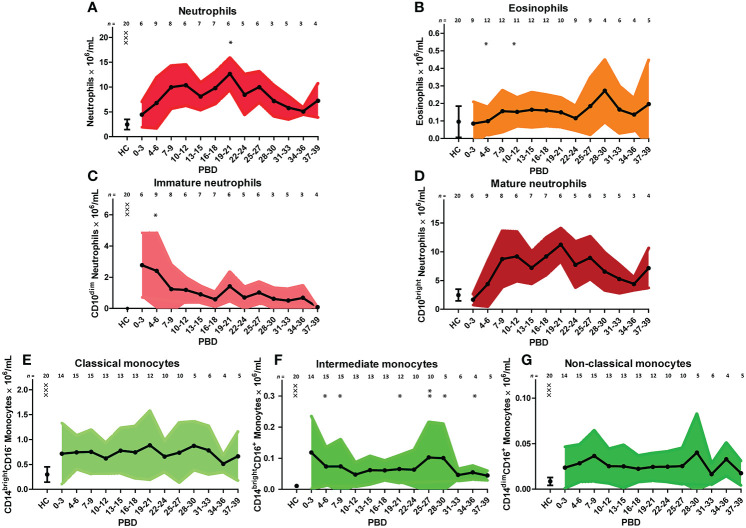
Supervised analysis of blood granulocyte and monocyte subsets after severe burn injury. Flow cytometry results of: **(A)** Neutrophils (CD15^+^CD16^+^ granulocytes). **(B)** Eosinophils (CD15^+^CD16^-^CD9^+^ granulocytes). **(C)** Immature neutrophils (CD10^dim^ neutrophils). **(D)** Mature neutrophils (CD10^bright^ neutrophils). **(E)** Classical monocytes (CD14^bright^CD16^-^ monocytes). **(F)** Intermediate monocytes (CD14^bright^CD16^+^ monocytes). **(G)** Non-classical monocytes (CD14^dim^CD16^+^ monocytes). Number of subjects per time interval is shown on top of the graphs. Values of burn wound patients and healthy controls (HC) are shown as mean (line and dots) ± standard deviation (colored band). Asterisks indicate significant differences in time within the burn patient group (linear mixed model analysis): *p < 0.05; **p < 0.01. Significant differences of outcomes in burn patients on PBD 0-3 compared to healthy controls are indicated by × (^×××^p < 0.001).

We verified the unsupervised findings by supervised flow cytometry analysis of data from all patients. The leukocyte increase after burn injury was indeed due to a rise in neutrophil numbers and was associated with shifts in maturation stage (**Figure 3A**). Eosinophil numbers (CD9^+^CD15^+^CD16^-^ granulocytes) increased over time but only to a small extent (**Figure 3B**). The high number of immature neutrophils at 0–3 days after injury decreased after PBD 6, but remained higher than in healthy controls until PBD 34–36. Mature neutrophil counts increased at PBD 4 and remained elevated from PBD 7 onward (**Figures 3C, D**). Supervised analysis confirmed the persistent increase in classical monocytes, but also revealed an increase in intermediate CD14^bright^CD16^+^ and non-classical CD14^dim^CD16^+^ monocytes. These data demonstrate that burn trauma induced a continuous release of (immature) neutrophils and monocyte subtypes.

### Burn Injury Induces an Increase in CCR4 and CCR6 Expressing CD4^+^ T Cells and Tregs From the Second Week After Injury Onward

**Figure 4:**
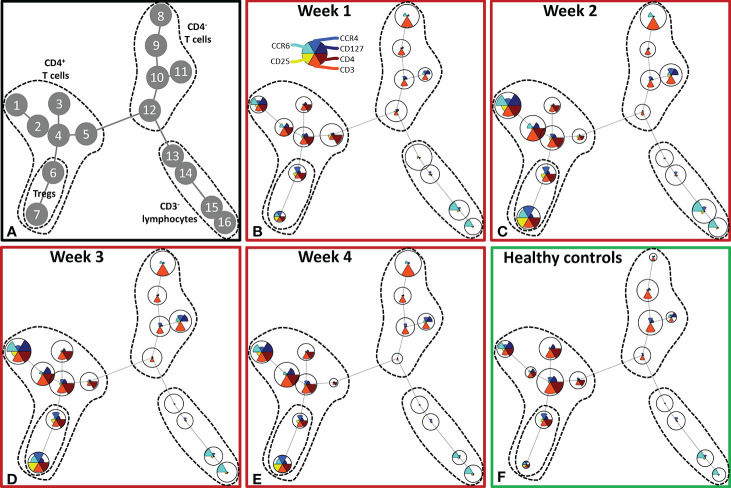
Unsupervised FlowSOM analysis of lymphocyte subtypes after severe burn injury. FlowSOM plots present proportions of populations and the expression of markers that were used in the lymphocyte flow cytometry panel (CD3, CD4, CD25, CD127, CCR4 and CCR6). **(A)** Cluster structure based on flow cytometry data of 10 healthy controls and 12 burn wound patients that were observed for 4 weeks. The most pronounced subtypes are encircled by dashed lines: CD4^+^ T cells (nodes 1-7), Tregs (nodes 6, 7), CD4^-^ T cells (nodes 8-12), CD3^-^ lymphocytes (nodes 13-16). FlowSOM plots of: **(B)** Week 1; **(C)** Week 2; **(D)** Week 3; **(E)** Week 4 after burn; **(F)** Healthy controls.

Although burn injury did not significantly alter the total number of lymphocytes, unsupervised analysis of the lymphocyte flow cytometry panel revealed changes in the T cell composition (**Figure 4**). Four main clusters of lymphocytes could be discriminated: CD4^+^ T cells (nodes 1–7), Tregs (nodes 6, 7), CD4^-^ T cells (nodes 8–12) and CD3^-^ lymphocytes (nodes 13–16) (containing B cells and NK cells) (**Figure 4A**). In the CD4^+^ T cell cluster, the CCR4^-^CCR6^-^ T cells (node 4), among which could be naïve T cells, decreased upon burn injury. CCR4^+^CCR6^+^ and CCR4^-^CCR6^+^ T cells (nodes 1 and 2, respectively) increased in week 2 and remained elevated in week 3 and 4. Two regulatory T cell populations were distinguished: CCR4^+^CCR6^-^ and CCR4^+^CCR6^+^ Tregs (nodes 6 and 7, respectively), which were both increased in week 2 and 3. In week 4, CCR4^+^CCR6^-^ Treg numbers were comparable to healthy controls, while the numbers of CCR4^+^CCR6^+^ Tregs were still increased. In the CD3^-^ lymphocyte cluster only small changes were observed.

**Figure 5:**
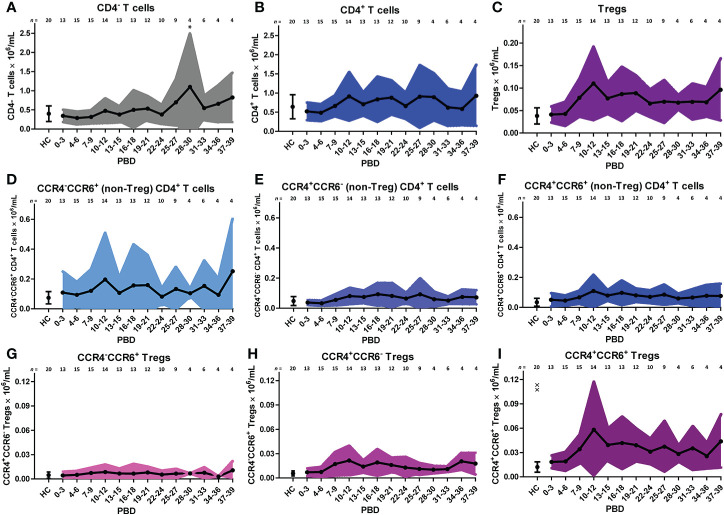
Supervised analysis of blood lymphocyte subsets after severe burn injury. Flow cytometry results of: **(A)** CD4^-^ T cells (CD3^+^CD4^-^ lymphocytes). **(B)** CD4^+^ T cells (CD3^+^CD4^+^ lymphocytes). **(C)** Tregs (CD3^+^CD4^+^CD25^+^CD127^-^ lymphocytes). **(D)** CCR4^-^CCR6^+^ CD4^+^ (non-Treg) T cells; **(E)** CCR4^+^CCR6^-^ CD4^+^ (non-Treg) T cells; **(F)** CCR4^+^CCR6^+^ CD4^+^ (non-Treg) T cells; **(G)** CCR4^-^CCR6^+^ Tregs; **(H)** CCR4^+^CCR6^-^ Tregs; **(I)** CCR4^+^CCR6^+^ Tregs. Number of subjects per time interval is shown on top of the graphs. Cell subset concentrations of burn wound patients and healthy controls (HC) are shown as mean (line and dots) ± standard deviation (colored band). Asterisks indicate significant differences in time within the burn patient group (linear mixed model analysis): *p < 0.05. Significant differences of outcomes in burn patients on PBD 0-3 compared to healthy controls are indicated by × (^××^p < 0.01).

Similar to the analysis of the innate cells, we took a supervised approach on the lymphocyte flow cytometry data of all patients to verify the unsupervised findings (**Figure 5**). The increase in CD4^+^ T cells in the second week after burn injury was confirmed, while the number of CD4^-^ T cells did not change (**Figures 5A, B**). A more detailed analysis showed that Treg numbers were increased from PBD 7 until 39 (**Figure 5C**). Also, we confirmed the increase in chemokine receptors (CCR4 and CCR6) expressing CD4^+^ T cells and Tregs (**Figures 5F, I**). Furthermore, we could confirm the increase in CCR4^+^CCR6^-^ Tregs (**Figure 5H**) after PBD 7, and observed a constant level of CCR4^-^CCR6^+^ CD4^+^ T cells (**Figure 5D**). We found more CCR4^+^CCR6^+^ CD4^+^ (non Treg) T cells than Tregs, suggesting that the balance might be tipped, enhancing the inflammation rather than resolving it. Thus, within the lymphocyte population, there was an increase in effector cells and Tregs from week 2 onward that show a mixed pro- and anti-inflammatory phenotype.

### Burn Injury Induces High Levels of Circulating Pro-Inflammatory Immune Mediators

**Figure 6:**
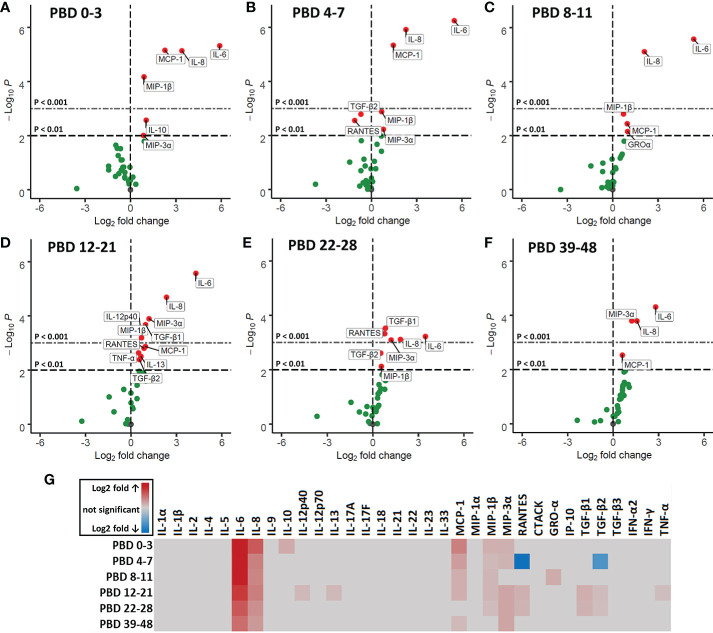
Volcano plots of 33 plasma immune factors after severe burn injury. Soluble mediators were analyzed in plasma of burn patients and healthy controls by Luminex immunoassay: MCP-1 (CCL2), MIP-1α (CCL3), MIP-1β (CCL4), MIP-3α (CCL20), GRO-α (CXCL1), IP-10 (CXCL10), IFN-α2, IFN-γ, TNF-α, TGF-β1, TGF-β2, TGF-β3, CTACK (CCL27), RANTES (CCL5), IL-1α, IL-1β, IL-2, IL-4, IL-5, IL-6, IL-8 (CXCL8), IL-9, IL-10, IL-12p40, IL-12p70, IL-13, IL-17A (CTLA-8), IL-17F, IL-18, IL-21, IL-22, IL-23, and IL-33 (NF-HEV). Differences between burn and healthy group were expressed as (Log2) fold change of healthy group (*n* = 13) on the x-axis and the (-Log10) p value on the y-axis of various time intervals after burn. **(A)** PBD 0 to 3 (*n* = 10 patients). **(B)** PBD 4 to 7 (*n* = 14 patients). **(C)** PBD 8 to 11 (*n* = 13 patients). **(D)** PBD 12 to 21 (*n* = 15 patients). **(E)** PBD 22-28 (*n* = 13 patients). **(F)** PBD 39 to 48 (*n* = 8 patients). Because of multiple testing, we considered a p value of < 0.01 to be significant. Black dashed line shows p = 0.01, gray dashed line shows p = 0.001, green dots indicate non-significant changes and red dots show significant changes. **(G)** Heatmap of significant (p < 0.01) fold changes compared to healthy controls (Log2 fold). Fold changes are shown in gray (not significant), red (increase) or blue (decrease).

To study circulating immune mediators induced by burn injury, we screened a broad panel of 33 cytokines, chemokines and growth factors in plasma of burn wound patients from PBD 0 until 48. To highlight significant changes in burn wound patients, data was transformed to fold changes in relation to the levels detected in healthy controls and presented in volcano plots (**Figures 6A–F**). Pro-inflammatory cytokines IL-6 and IL-8 were increased at all time intervals. Furthermore, we found an increase in chemokines MCP-1 (CCL2), MIP-1β (CCL4), RANTES (CCL5) and MIP-3α (CCL20), which are known chemoattractants for monocytes, granulocytes and T cells during inflammation (27). IL-10 levels were only increased at PBD 0–3. RANTES and TGF-β2 were decreased at PBD 4–7 and increased at PBD 12–28, following a similar pattern as the number of thrombocytes after burn injury (**Supplementary Figure 2C**). A summary of the significant increases and decreases in soluble factors is presented in a heatmap (**Figure 6G**).

**Figure 7:**
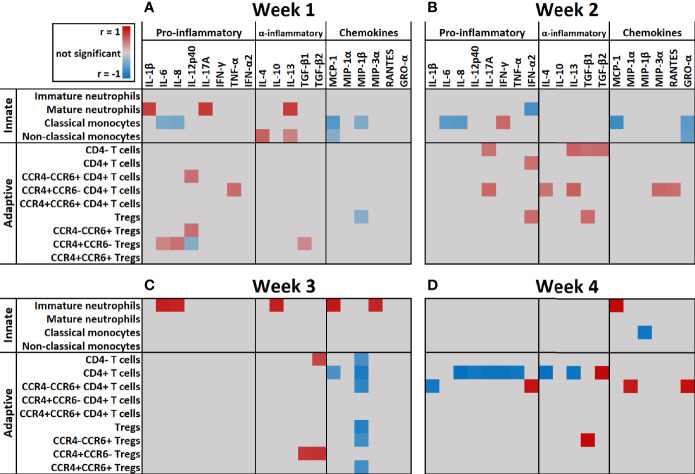
Heatmap of correlation coefficients of immune cells and soluble mediators over time. Significant (p < 0.05) correlation coefficients (r) of immune cell counts and fold change of soluble mediators at: **(A)** Week 1 (*n* = 13 patients); **(B)** Week 2 (*n* = 15 patients); **(C)** Week 3 (*n* = 10 patients); **(D)** Week 4 (*n* = 5 patients) after burn injury. Correlations were measured by Pearson tests and results are shown in gray (not significant), red (positive correlation) or blue (negative correlation).

Next, we correlated immune cell subset numbers to the fold changes of soluble mediators by Pearson’s correlation coefficient tests and visualized the significant (p < 0.05) correlations in a heatmap (**Figure 7**). In the first week after injury, the most pronounced positive correlations were found between mature neutrophils and IL-1β, IL-13 and IL-17A (r > 0.7; p < 0.001) and between non-classical monocytes and IL-4 (r > 0.5; p < 0.001). In the first and second week, there were negative correlations between classical monocytes and IL-6, IL-8, MCP-1 and GRO-α (r between -0.4 and -0.7; p < 0.001). Immature neutrophils showed a strong positive correlation in the third week with IL-6, IL-8, IL-10, MCP-1 and MIP-3α and in the fourth week with MCP-1 (r > 0.8; p < 0.001). In the first 2 weeks after injury, there were only weak positive correlations between T cells and soluble mediators, but this pattern changed in week 3 and 4, where strong correlations were predominantly found between T cells and immune mediators. In week 3, CD4^+^ T cells and Tregs showed strong negative correlations with MIP-1β (r < -0.8; p < 0.001) and CCR4^+^CCR6^-^ Tregs showed a strong positive correlation with TGF-β1 and TGF-β2 (r > 0.7; p < 0.001). Four weeks after injury, we observed positive correlations between CCR4^-^CCR6^+^ CD4^+^ T cells and MIP-1α, GRO-α and IFN-γ (r > 0.8; p < 0.001) and strong negative correlations between CD4^+^ T cells and IL-4, IL-8, IL-12p40, IL-13, IL-17A, IFN-γ and TNF-α (r < -0.9; p < 0.001). Seemingly, in week 1–3 the presence of innate immune cells can be linked to pro-inflammatory cytokines, while in week 3–4 most pronounced correlations were found between CD4^+^ T cell subsets and specific mediators (**Figure 7**).

## Discussion

Here, we performed a longitudinal study on 20 severely burned patients and investigated the effects of severe burn injury on the systemic immune response. We reveal that upon burn injury there is an immediate surge of innate immune cells, with initially a large contribution of immature neutrophils, but no increase in lymphocyte numbers. These cellular responses could not be correlated to the patient’s TBSA, which might indicate maximum response levels. Notably, only patients submitted to the ICU were included in this study. Simultaneously with the cellular influx, increased levels of various pro-inflammatory cytokines were found. This innate immune and cytokine response decreased to some extent over time, but persisted for at least three weeks. From the second week onward, shifts in T cell subpopulations were observed: within the T cell population, there was an increase of CCR4 and CCR6 expressing cells and although Treg numbers increased as well, the overall phenotype of the CD4^+^ T cells and Tregs appeared to be rather pro-inflammatory than anti-inflammatory.

The increase in granulocytes could mainly be attributed to neutrophils, and within this population both mature and immature neutrophils were increased. In the first week after injury, mature neutrophil counts correlated with IL-17A, which is known to accelerate neutrophil recruitment (28). Moreover, levels of IL-6 and IL-8 were increased over the complete study period and could be correlated to the number of immature neutrophils in the third week post injury. IL-6 and IL-8 are involved in neutrophil recruitment and chemotaxis (28, 29). In a healthy situation, immature neutrophils are usually absent in the circulation as neutrophils normally mature within the bone marrow before they are released to the bloodstream (30). The early release of immature neutrophils can be caused by an emergency response of the immune system to acute inflammation, such as trauma, burn or sepsis (26, 31, 32). During acute inflammation, neutrophils produce ROS, elastase, myeloperoxidase and release neutrophil extracellular traps (NETs) which can damage tissues by their cytotoxicity and can cause ischemia through thrombocytosis (10, 33). In other studies of patients with large burn injuries (>15% TBSA), expression of CD11b on neutrophils was increased in the first week while expression of CD16 was reduced (34, 35). Other studies also reported a fast decrease of CD16 expression due to the immaturity of neutrophils (36, 37). We also observed reduced CD16 expression on immature neutrophils (data not shown). It has been shown that continuous release of neutrophils into the circulation can lead to bone marrow exhaustion that in turn can lead to compromised innate immunity (38–40). Although literature on the functions or activities of immature neutrophils is conflicting, some papers state that immature neutrophils are underdeveloped and that high numbers and their active state might induce tissue damage causing secondary progression of the burn injury (13, 38, 39). Other studies have shown that trauma-induced immature neutrophils in blood of patients with systemic inflammation actually show decreased chemotactic activity and increased life-span, and therefore reside longer in the bloodstream then mature neutrophils (41–43). Also, it was shown that immature neutrophils have a reduced oxidative burst and phagocytic activity and that they are less potent in supporting innate immune defenses (43–45). Another study showed that the reduced oxidative burst in neutrophils can last for up to 3.5 months (46). It is however still unclear whether immature neutrophils are overall beneficial or detrimental for wound healing (47). Nevertheless, our data demonstrate that burns can cause a long-lasting presence of both immature and mature neutrophils that may be harmful for wound healing, distant organs and survival.

During wound healing, the first cells attracted to the site of injury are neutrophils, followed by monocytes, which upon arrival differentiate into macrophages or dendritic cells (48). In response to acute systemic inflammation, the bone marrow releases its reserve of classical monocytes into the bloodstream to replace the monocytes that migrated into inflamed tissue (49). CD14, a co-receptor of various Toll-like receptors, is increased on monocytes upon burn injury and helps to detect bacteria in the body (43, 50). We found increased numbers of all three monocyte subtypes in blood, while classical monocytes were the most prevalent. Classical monocytes mainly exert pro-inflammatory functions and can become monocyte-derived macrophages or dendritic cells upon infiltration of inflamed tissue (51). Negative correlations were found between classical monocytes and MCP-1, a known chemoattractant for monocytes (52). Binding of MCP-1 to CCR2 on circulating monocytes might have resulted in lower levels of free MCP-1 (53, 54). Alternatively, MCP-1 might have induced migration of classical monocytes toward affected tissues (52). In this cohort of burn patients, the number of classical monocytes remained elevated for at least 39 days. Other studies on burn patients also found increased levels of classical monocytes during systemic inflammation (55). Non-classical monocytes, that are described as more anti-inflammatory monocytes are thought to acquire the pro-healing macrophage phenotype (M2) in the injured tissue (53). Although we found an increase in this monocyte subtype upon burn injury as well, their numbers were much lower than that of classical monocytes. Taken together, this might indicate that the balance of monocyte phenotypes is shifted toward a pro-inflammatory, rather than an anti-inflammatory state, that persists for weeks after burn injury.

Later in the posttraumatic immune response (days to weeks), lymphocytes arrive at the site of injury to regulate the inflammation and support tissue restoration together with pro-healing macrophages (56). To our knowledge there is no information on the dynamics of T cell activation and differentiation after burn injury in humans. Here, we established that while the number of lymphocytes in blood was largely unaffected upon burn injury, the T cell subset composition was altered from the second week after injury, indicative of an adaptive immune response (57). To study the phenotype of circulatory T cells in burn patients, we analyzed CCR4 and CCR6 expression and found increased numbers of CCR4^+^CCR6^+^ CD4^+^ T cells, which might indicate a shift toward a Th17 T cell phenotype. This notion was supported by increased levels of MIP-3α, a natural ligand of CCR6 (58), from PBD 12 onward, as well as high levels of IL-6, TGF-β1, and TGF-β2, which in combination can induce a Th17 response (18, 59). Additionally, we observed increased numbers of CCR4^+^CCR6^-^ CD4^+^ T cells, indicative of a Th2 phenotype (60). This was associated with an increase in IL-13, a Th2 cytokine (61), at PBD 12–21. Animal experiments have also shown that burn injury induces a mixed Th2/Th17 response. Moreover, IL-17 which is released by Th-17 cells is involved in the recruitment and activation of neutrophil (18, 62, 63). This might explain the high neutrophil counts that peak during PBD 16–21. Burn injury was also associated with an increase of Tregs, which are likely part of the immune system’s attempt to resolve the acute inflammation (64). Upon *in vitro* culture, Treg from severely burned patients produced elevated levels of IL-10 in the first 21 days after injury (65). Here, plasma levels of IL-10 were only increased at PBD 0–3. An early increase of serum IL-10 was also found in severely burned children, which was followed by a small, non-significant elevation of circulatory IL-10 afterward (5, 66, 67). This suggests that *in vivo*, the suppressive response from Tregs might be impaired after PBD 3, possibly due to the high levels of pro-inflammatory cytokines and number of acute phase immune cells. In addition, we found evidence for Treg differentiation, as both CCR6^+^ and CCR6^-^ Tregs were present. This suggests that there is a burn-induced mixed phenotype within the Treg population (58, 68). The transformation of Tregs into putative pathophysiologic Tregs has been proposed before (69, 70) and, in this case, could be caused by burn-induced DAMPs and pro-inflammatory mediators such as the CCR6 ligand MIP-3α. Although functional assays are needed to verify the phenotype of these Tregs, our data suggest that severe burn injury causes a shift in the T cell subsets toward more pro-inflammatory subtypes, tipping the balance and thereby continuing the inflammation.

The increase in inflammatory mediators is indicative of a persistent systemic inflammatory immune response due to severe burns. However, all included burn patients were at high risk for infection, like central line-associated bloodstream infections and bloodstream infections" should be changed to "at high risk for infection, such as central line-associated bloodstream infection. These infections were not observed in this study but bacterial presence could have influenced the levels of inflammatory mediators. Medication could have affected the immune response, but all patients were treated in a similar manner, involving the administration of antibiotics and analgesics. Although the differences in immune components in the blood between burn wound patients and healthy controls were significant and remained increased over time, the sample size is a limitation of our study. Missing data was caused by less frequent blood withdrawal at the infirmary, delayed start of study participation, patient discharge and death. In addition, we acknowledge that the difference in age and gender between burn patients and healthy controls represents a limitation of this work, as aging and gender can affect the immune response (71, 72). Unfortunately, the group size was insufficient to analyze these differences in more detail. Supervised gating of flow cytometry data can be challenging due to biological variation and the fact that manual gating relies on the researcher’s prior knowledge causing bias in the analysis (73). To overcome this, we took a combined approach of both supervised and unsupervised analysis of our data, and showed that they largely reached the same outcome. To better understand the behavior of these immune cells, it would be interesting to study functionality of these cells after burn injury. Furthermore, we are curious to see if the observed systemic immune response is reflected by the local immune response to burn injury and will pursue this aim in the near future.

Information on the immunological mechanisms driving burn-induced inflammation and pathophysiology is very limited. Because of the excessive and persistent inflammation, it could be beneficial for burn wound patients to use anti-inflammatory drugs (74). Directed therapy that either decreases the influx of neutrophils or supports the suppressive arm of the immune system, might lower the risk of complications caused by the systemic inflammation, which in turn should improve wound healing. Because of variation between burn wounds, patients and differences in the intensity of the burn-induced immune response, treatment should be empirical and personalized to improve the outcome.

Taken together, we showed that the burn-induced leukocytosis is mainly due to an increase of neutrophils and monocytes and that burn injury caused a long-lasting influx of immature neutrophils. The persistent elevated levels of pro-inflammatory cytokines and the shifts in neutrophil and lymphocyte composition suggest that the immune system remains in a long-term pro-inflammatory state rather than switching to a resolving state. Because these immune reactions are likely to strengthen one another and keep the inflammation going, we need to search for ways to resolve inflammation in an early stage in order to improve burn treatment, prevent secondary complications, and reduce length of hospital stay.

## Data Availability Statement

The raw data supporting the conclusions of this article will be made available by the authors, without undue reservation.

## Ethics Statement

The studies involving human participants were reviewed and approved by the Medical Ethics Committee of the VU Medical Center, Amsterdam, Netherlands. The patients/participants provided their written informed consent to participate in this study.

## Author Contributions

MU, IJ, and HK conceived the study. MS and AP recruited patients and collected samples and subject data. PZ and EJ were responsible for patient care and interpretation of clinical data. PM and MV performed experiments. PM, MV, BB, BC, and AP were involved in data analysis. PM wrote the manuscript. MV, BB, HK, MU, and IJ assisted on writing of the manuscript. MS, AP, PZ, and EJ contributed to revision of the manuscript. All authors contributed to the article and approved the submitted version.

## Funding

Funding was provided by the Dutch Burn Foundation under project number WO/17.108.

## Conflict of Interest

The authors declare that the research was conducted in the absence of any commercial or financial relationships that could be construed as a potential conflict of interest.

## Supplementary Material

Supplementary Figure 1 Gating strategy for supervised flow cytometry analysis. Gating strategy is shown for all three panels.

Supplementary Figure 2 Levels of systemic inflammation indicators in blood of burn patients. Laboratory results of: **(A)** Serum CRP levels compared to healthy reference values (dotted lines: 0-10 mg/L). **(B)** Serum albumin levels compared to healthy reference values (dotted lines: 34-54 g/L). **(C)** Blood thrombocyte counts compared to healthy reference values (dotted lines: 150-400 × 10^9^/L). **(D)** Number of patients per time point in A-C. Values of burn wound patients are shown as mean (line and dots) ± standard deviation (colored band).

Supplementary Table 1 Demographics and sampling of subjects. A) Demographic data is shown per patient and per group. For the burn patients the age range was 18-82 and for the healthy controls this was 23-62. B) Black dots show frequency of blood sampling of individual burn patients. For the analyses, multiple measures of one patient within one time interval were averaged.

Supplementary Table 2 Antibodies used for flow cytometry. All antibodies were purchased at Miltenyi Biotec GmbH, Bergisch Gladbach, Germany.
