# Supplementary figures and images for: Persistent Systemic Inflammation in Patients With Severe Burn Injury Is Accompanied by Influx of Immature Neutrophils and Shifts in T Cell Subsets and Cytokine Profiles
Source: Front Immunol. 2021 Jan 29;11:621222. doi: 10.3389/fimmu.2020.621222 (PMC7879574; doi:10.3389/fimmu.2020.621222)

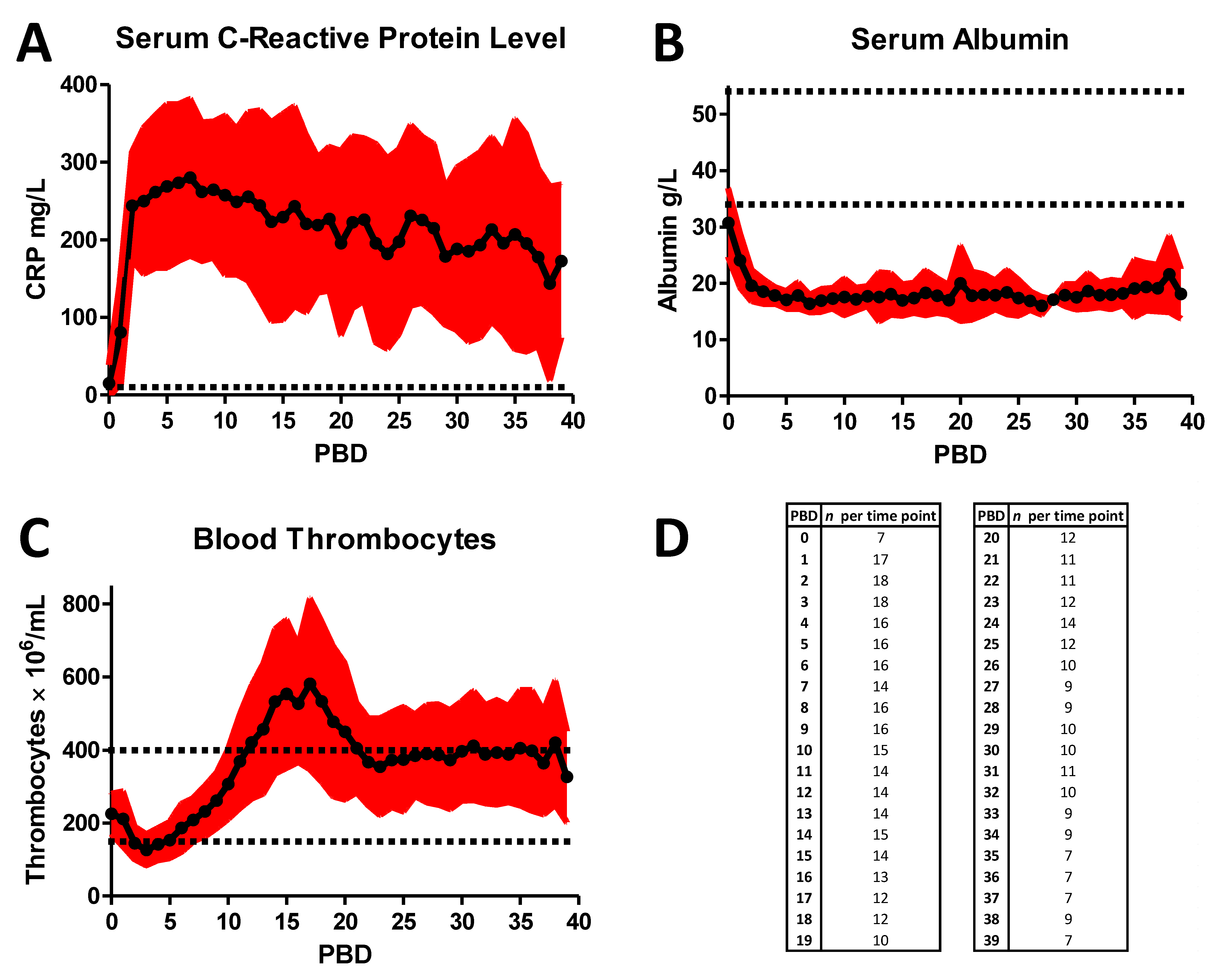

Supplement: Supplementary Figure 1 — Gating strategy for supervised flow cytometry analysis. Gating strategy is shown for all three panels. [file Image_1.tiff]

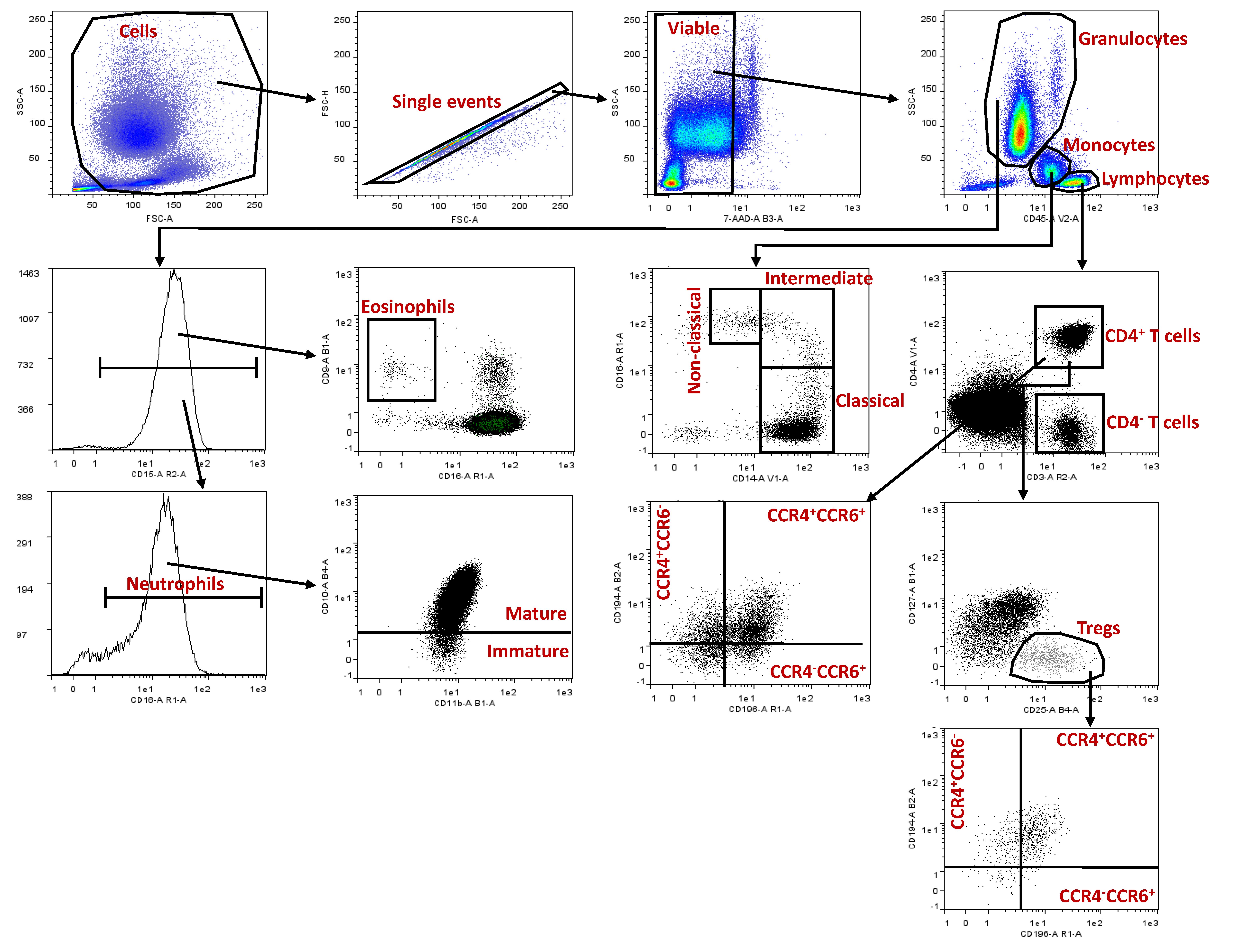

Supplement: Supplementary Figure 2 — Levels of systemic inflammation indicators in blood of burn patients. Laboratory results of: (A) Serum CRP levels compared to healthy reference values (dotted lines: 0-10 mg/L). (B) Serum albumin levels compared to healthy reference values (dotted lines: 34-54 g/L). (C) Blood thrombocyte counts compared to healthy reference values (dotted lines: 150-400 × 109/L). (D) Number of patients per time point in A-C. Values of burn wound patients are shown as mean (line and dots) ± standard deviation (colored band). [file Image_2.tiff]
