# Supplementary material for: Persistent Systemic Inflammation in Patients With Severe Burn Injury Is Accompanied by Influx of Immature Neutrophils and Shifts in T Cell Subsets and Cytokine Profiles
Source: Front Immunol. 2021 Jan 29;11:621222. doi: 10.3389/fimmu.2020.621222 (PMC7879574; doi:10.3389/fimmu.2020.621222)

# SUPPLEMENTAL TABLES

**Supplemental Table 1. Demographics and sampling of subjects.** **A)** Demographic data is shown per patient and per group. For the burn patients the age range was 18-82 and for the healthy controls this was 23-62. **B)** Black dots show frequency of blood sampling of individual burn patients. For the analyses, multiple measures of one patient within one time interval were averaged.


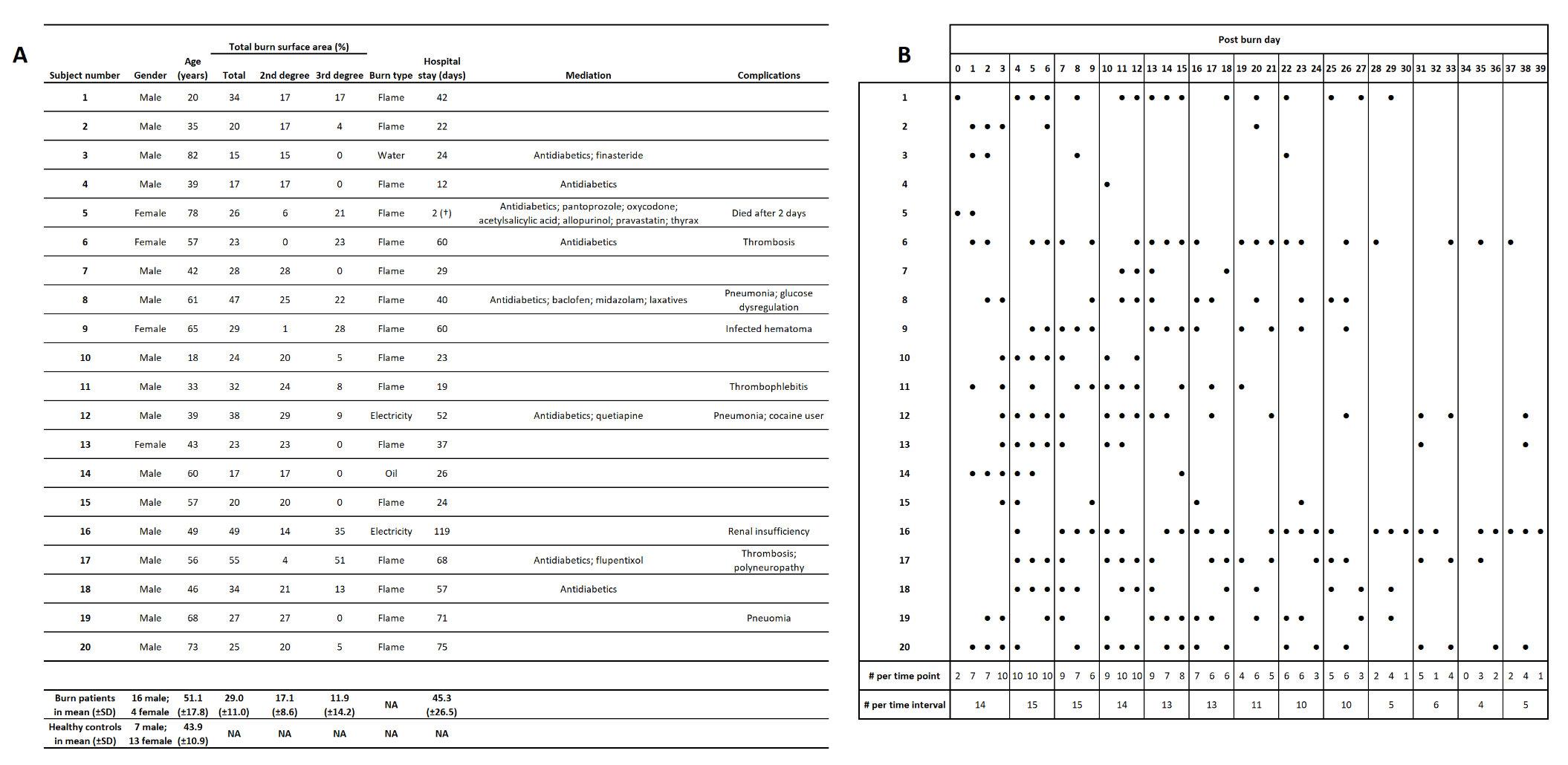


**Supplemental Table 2. Antibodies used for flow cytometry.** All antibodies were purchased at Miltenyi Biotec GmbH, Bergisch Gladbach, Germany.


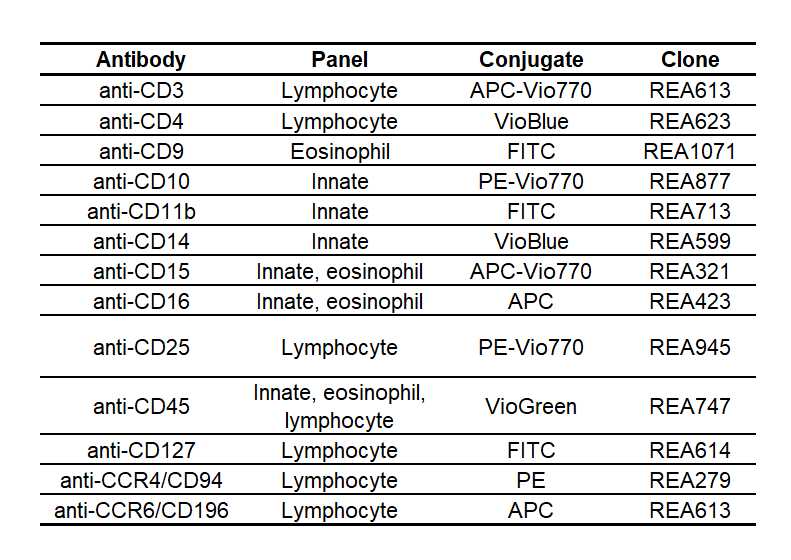

Supplement: Supplementary Table 1 — Demographics and sampling of subjects. A) Demographic data is shown per patient and per group. For the burn patients the age range was 18-82 and for the healthy controls this was 23-62. B) Black dots show frequency of blood sampling of individual burn patients. For the analyses, multiple measures of one patient within one time interval were averaged. [file Table_1.docx]
